# A learning based framework for diverse biomolecule relationship prediction in molecular association network

**DOI:** 10.1038/s42003-020-0858-8

**Published:** 2020-03-13

**Authors:** Zhen-Hao Guo, Zhu-Hong You, De-Shuang Huang, Hai-Cheng Yi, Zhan-Heng Chen, Yan-Bin Wang

**Affiliations:** 1 The Xinjiang Technical Institute of Physics and Chemistry, Chinese Academy of Sciences, 830011 Urumqi, China; 2 University of Chinese Academy of Sciences, 100049 Beijing, China; 3 Computer Science Department, Tongji University, 200000 Shanghai, China; 4 School of Cyber Science and Technology, Zhejiang University, 310000 Hangzhou, Zhejiang China

**Keywords:** Bioinformatics, Biological models, Computational biology and bioinformatics, Data mining

## Abstract

Abundant life activities are maintained by various biomolecule relationships in human cells. However, many previous computational models only focus on isolated objects, without considering that cell is a complete entity with ample functions. Inspired by holism, we constructed a Molecular Associations Network (MAN) including 9 kinds of relationships among 5 types of biomolecules, and a prediction model called MAN-GF. More specifically, biomolecules can be represented as vectors by the algorithm called biomarker2vec which combines 2 kinds of information involved the attribute learned by k-mer, etc and the behavior learned by Graph Factorization (GF). Then, Random Forest classifier is applied for training, validation and test. MAN-GF obtained a substantial performance with AUC of 0.9647 and AUPR of 0.9521 under 5-fold Cross-validation. The results imply that MAN-GF with an overall perspective can act as ancillary for practice. Besides, it holds great hope to provide a new insight to elucidate the regulatory mechanisms.

## Abstract (web summary)

Guo et al. construct a large scale Molecular Associations Network (MAN) including 9 kinds of associations among 5 types of biomolecules, namely protein, miRNA, lncRNA, disease and drug. They further propose a computational model, MAN-GF, that can predict links between these biomolecules and displays a substantial performance under 5-fold cross validation.

## Introduction

The central rule proposed by Crick F. et al. explains the flow of genetic information in living organisms and directs the development of molecular biology for decades^1^. However, accumulated evidence reveals the existence of different kinds of biomolecules in human cells and proves that the relationships between them are fundamental in cellular processing, information transfer and decision-making^2,3^. For instance, with the introduction of the competing endogenous RNAs (ceRNA) mechanism, more and more experiments and literatures indicate that the interaction of ncRNA and mRNA regulates gene expression^4^. Cumulative studies have indicated that a series of ncRNAs are associated with numerous diseases such as cancers^5^, blood diseases^6^, and neurodegeneration diseases^7^. Consequently, microscopic study of the relationships between biomolecules not only opens innovative insights to understand life process, but also facilitates to disease prevention, diagnosis, treatment, and drug development.

Benefiting from the development of high-throughput technologies, vast array of sequences and relationships are determined and published on numerous online databases such as HMDD^8^, STRING^9^, and DrugBank^10^. Although experimental verification-based methods have strongly promoted people’s understanding of cellular activities at the molecular level. The number of relationships that are validated by these experiments only occupies a small part of the whole. Moreover, the high false-positives and false-negatives presented in manual experiments due to various factors may moderate and mislead the progression^11^. It is necessary and urgent to propose reliable and efficient computational approaches to handle massive data for the guidance of practical experiments.

In fact, numerous computational prediction methods have been designed to infer new relationships between transcripts, translations and small molecule compounds over the past few years. Most prediction models belong to several categories owing to research object or calculation methods. According to research object, the prediction model can be segmented into the following typical representatives. For protein–protein interaction (PPI), Huang et al. proposed a sequence information based model to predict potential interaction by using weighted sparse representation model combined with global encoding^12^. For ncRNA-protein (RPI), Yi et al. achieved outstanding prediction results on multiple RIP datasets by combining evolutionary information and deep learning frameworks^13^. For ncRNA-disease, Guo et al. presented a learning-based method to predict uncovered lncRNA-disease associations by integrating multiple types biological information and rotation forest^14^. For miRNA-disease, Li et al. predicted potential associations by Network Topological Similarity Based on DeepWalk^15^. According to calculation manner, the prediction model can be divided into network-based methods, machine learning-based methods, and matrix decomposition-based methods. A supervised framework was proposed by Wang et al. to predict protein–protein interactions through combining stacked sparse autoencoder and probabilistic classification vector machine (PCVM) classifier^16^. Li et al. introduced a matrix decomposition-based method called MCMDA to predict potential associations by updating the adjacency matrix of miRNA-disease^17^. Huang et al. came up with a network-based model called EPLMI to discover potential miRNA-lncRNA interaction based on two-way diffusion from expression profiles^18^.

Recently, the discovery of new kinds of biomolecules and the evidence of adequate experimentally validated relationships inspire researchers to take extra biomarker as a bridge or intermediary to improve the performance of the computational model. Chen et al. took lncRNA as an intermediary to discover potential miRNA-disease associations in heterogeneous networks through label propagation algorithms^19^. Peng et al. characterized the similarity between miRNA-gene and disease-gene, respectively, and predicted the association between miRNA-disease in the framework of machine learning model^20^. The methods proposed by them mitigates the impact of data loss on predictions to a certain extent and integrates the idea of the pathway. However, they are still the congeners of reductionism essentially.

Reductionism, which disassembles the biological system into several basic components based on composition or function from a modular point of view and studies each unit in a focused or isolated manner, has been the dominant idea in bioinformatics for decades^21^. Given the fundamental principle plays in network biology and the increasingly clear evidence shed light on that cells, as inherent in a complete individual, are affected by constituent elements without a doubt. Different kinds of biomolecules and relationships are like nodes (biomolecules) and edges (relationships) in a network (cell). Network is an unstructured data that is common in the real world and is widely studied. Modeling cells into networks is compatible and can be borrowed from existing efficient computer network algorithms. To address this challenge, the large-scale Molecular Associations Network (MAN) is constructed by various kinds of relationships among several different types of biomolecules.

**Fig. 1:**
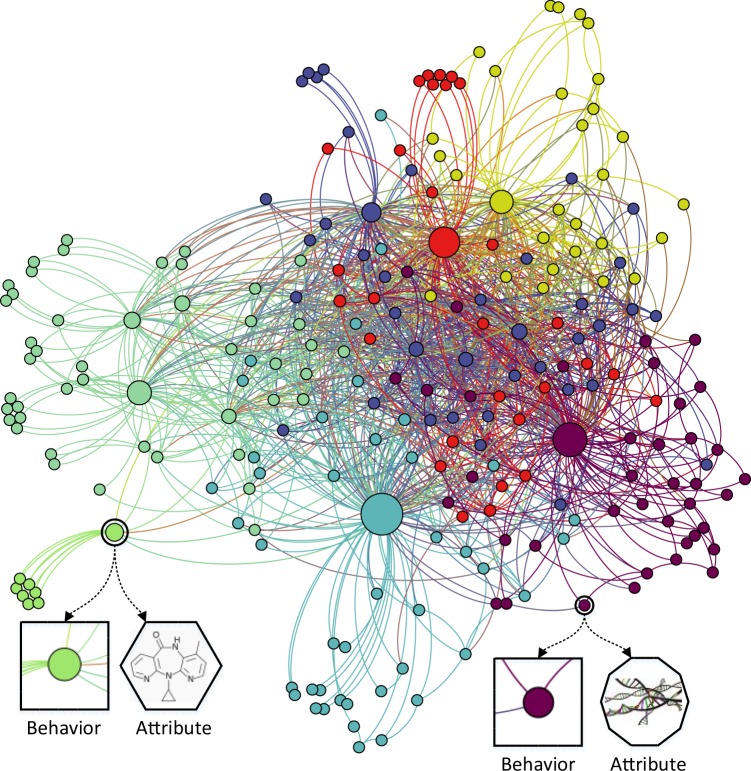
Schematic diagram of molecular associations network. Different colored nodes represent different types of biomolecules. As shown in the figure, each node can be described or represented by two kinds of feature including the node behavior (relationships with other nodes) and the node attribute (e.g., RNA sequences, disease semantics, and drug chemical structure).

In this paper, we construct a network called MAN including nine kinds relationships among five types biomolecules and a model called MAN-GF that can predict any edges between arbitrary nodes in the framework. The network is shown as Fig. 1. Firstly, relationships including lncRNA-miRNA, drug-disease, protein–protein, etc. between biomolecules such as protein, ncRNA and disease, are collected from diverse databases to develop the network. After MAN is defined as a homogenous undirected graph, we construct a lower triangular portion of adjacency matrix to facilitate storage and computation. Secondly, each node in MAN can be represented by the algorithm called biomarker2vec by combining two kinds of feature including the node behavior (edges with other nodes) learned by GF and the node attribute (e.g., ncRNA sequences, disease semantics, and drug molecular fingerprint) learned by k-mer etc. Finally, Random Forest, a common ensemble classifier, is applied to perform training and prediction tasks based on known positive samples and randomly drawn negative samples. Note that in each step, parameters are set to default values to improve the reproducibility of the experiment. The proposed MAN-GF model achieves remarkable results with AUC of 0.9647 and AUPR of 0.9521 under 5-fold Cross-validation, respectively. Moreover, the feature comparison experiment indicates that vectors integrated two kinds of feature are more distinguishable. Furthermore, experiments on different proportions of training sets proves that MAN-GF can still achieve satisfactory prediction effects even if the training samples are rare. In addition, we make a special test to compare the traditional method based on local idea and the proposed model based on global view. Results demonstrate that the MAN do contain a wealthy of biology information and promoted the prediction performance from the comprehensive perspective with regards to isolated view. Finally, a miRNA-disease association prediction case study strongly proves the effectiveness of the proposed framework in actual environments. In short, we detect how biomolecules interact in human cells by integrating traditional multi-type biology data and the state of art complex network technologies from a systemic perspective. There is no doubt that the development of reliable global view to assist in solving biological problems will have a revolutionary impact on current bioinformatics research. MAN-GF will become the vital engine of detecting undiscovered relationships and we hope this work can bring beneficial inspiration and advance for related network biology and biomedical research.

## Results

### Multi-type relationship prediction under 5-fold Cross-validation

In relationship prediction, a network with a certain fraction of edges removed is given, and we want to predict the missing edges. Here, Cross-validation is applied to assess the link prediction effect of the model globally. Cross-validation is a commonly used standard for evaluating the performance of machine learning models. Under the 5-fold Cross-validation, all biomolecular relationships are randomly divided into five mutually exclusive subsets of approximately equal size. Each subset can be treated as the test set to assess the performance of the model, and the remaining subsets will be regarded as training set to construct the classifier.

Receiver Operating Characteristic Curve (ROC) is a curve drawn on the abscissa False Positive Ratio (FPR) and the ordinate True Positive Ratio (TPR) as the coordinate axes. The horizontal and vertical axes of the Recall Precision (PR) curve are Recall and Precision, respectively. The surface enclosed by the curve and the coordinate axis, called AUC or AUPR, can objectively reflect the classification performance of the model to some extent. For 5-fold Cross-validation, MAN-GF has achieved AUC of 0.9647 and AUPR of 0.9521.

In order to evaluate our model fairly and broadly, a range of evaluation criteria including accuracy (Acc.), sensitivity (Sen.), specificity (Spec.), precision (Prec.), and MCC were used to objectively and comprehensively describe the predictive performance. Under 5-fold Cross-validation, the results are shown in Table 1, in which the average values of accuracy (Acc.), sensitivity (Sen.), specificity (Spec.), precision (Prec.) and MCC were separately 91.47, 90.96, 91.98, 91.90, 82.94, and 96.47, the standard deviations of the above data were 0.22, 0.32, 0.25, 0.24, 0.44, and 0.13, respectively. The competitive results in the indicators demonstrate competitive predictive power of the proposed model, and the lower standard deviation prove that the framework exhibits stability and robustness in various environments.

**Table 1:** Multi-type relationship prediction results of various evaluation criteria under 5-fold Cross-validation.

fold	Acc. (%)	Sen. (%)	Spec. (%)	Prec. (%)	MCC (%)	AUC (%)
0	91.35	90.71	92.00	91.89	82.71	96.38
1	91.39	91.16	91.62	91.58	82.78	96.41
2	91.86	91.41	92.31	92.24	83.72	96.69
3	91.38	90.68	92.07	91.96	82.76	96.46
4	91.37	90.84	91.89	91.81	82.74	96.41
Average	91.47 ± 0.22	90.96 ± 0.32	91.98 ± 0.25	91.90 ± 0.24	82.94 ± 0.44	96.47 ± 0.13

### Feature importance comparison

As mentioned in the introduction, each biomolecule in the entire network can be described by two kinds of feature including biomolecule attribute and behavior. In this chapter, we will elaborate on the impact of each type of information on relationship prediction tasks between biomolecules in the MAN.

The results of different models under 5-fold Cross-validation including pure attribute-based methods, pure behavior-based methods, and method of combining the above two kinds of information are shown in the Table 2. After combining different types of feature, it is obvious that the representation vector of the node has more prominent characteristic expression ability and is easier to distinguish.

**Table 2:** Comparison of various evaluation criteria based on different types of feature including pure attribute, pure behavior, and combination of above two.

Feature	Acc. (%)	Sen. (%)	Spec. (%)	Prec. (%)	MCC (%)	AUC (%)
Attribute (A)	87.92 ± 0.30	90.44 ± 0.11	85.40 ± 0.54	86.10 ± 0.45	75.94 ± 0.59	93.76 ± 0.26
Behavior (B)	89.75 ± 0.25	87.69 ± 0.39	91.82 ± 0.33	91.47 ± 0.31	79.58 ± 0.50	95.34 ± 0.16
Both	91.47 ± 0.22	90.96 ± 0.32	91.98 ± 0.25	91.90 ± 0.24	82.94 ± 0.44	96.47 ± 0.13

### Comparison performance based on varying proportion of training set

In order to explore the effect of varying proportion of training set on the prediction performance, we extracted different percentage of edges of the whole network to obtain the representation vectors by network embedding. Specifically, we take 20, 40, 60, and 80% of all edges as known samples, and map nodes to vectors through GF. When performing link prediction tasks, the training set is the known edges, and the test set is the remaining edges i.e., 80, 60, 40 and 20% of the total edges. Each node is represented as a 64-dimensional vector which processed by only GF. The results are in the Table 3.

**Table 3:** Comparison of various evaluation criteria based on varying number of training samples.

Percentage	Acc. (%)	Sen. (%)	Spec. (%)	Prec. (%)	MCC (%)	AUC (%)
20%	78.03	73.56	82.51	80.79	56.29	85.61
40%	84.56	80.90	88.21	87.28	69.30	91.52
60%	87.27	84.34	90.19	89.58	74.66	93.67
80%	89.78	87.60	91.95	91.59	79.63	95.39

### Classifier comparison

**Fig. 2:**
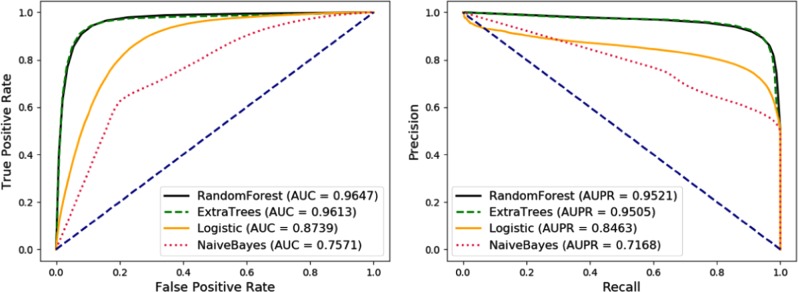
Comparison of the receiver operating characteristic curves (ROC), area under ROCs, precision recall (PR) curves, and area under PRs based on different classifiers including random forest, extra tree, logistic regression and naive bayes. These classifiers achieved corresponding AUCs of 0.9647, 0.9613, 0.8739, and 0.7571, and corresponding AUPRs of 0.9521, 0.9505, 0.8463, and 0.7168.

The performance of classifiers is different on different datasets. In this section, we compare the performance of different classifiers including Random Forest (RF), Extra Trees (ET), Logistic Regression (LR), and Naive Bayesian (NB) on MAN and try to analyze the reasons. Under 5-fold Cross-validation, the results are shown in Table 4 and Fig. 2. Note that all classifiers were adopted from Scikit-learn library and all parameters were set to default values.

**Table 4:** Comparison of various evaluation criteria based on different classifiers including Naive Bayes, Logistic Regression, Extra Tree and Random Forest under 5-fold Cross-validation.

Classifier	Acc. (%)	Sen. (%)	Spec. (%)	Prec. (%)	MCC (%)	AUC (%)
NB	67.69 ± 2.32	77.47 ± 2.12	57.91 ± 3.01	64.81 ± 2.07	36.07 ± 4.68	75.71 ± 1.55
LR	80.78 ± 0.44	85.16 ± 1.07	76.41 ± 0.34	78.30 ± 0.19	61.81 ± 0.94	87.39 ± 0.28
ET	91.77 ± 0.17	91.05 ± 0.22	92.50 ± 0.13	92.39 ± 0.14	83.56 ± 0.34	96.13 ± 0.04
RF	91.47 ± 0.22	90.96 ± 0.32	91.98 ± 0.25	91.90 ± 0.24	82.94 ± 0.44	96.47 ± 0.13

The cause of this phenomenon can be explained as follows. For Naive Bayesian, the representation vector may not be independent between degrees, which is contrary to the hypothesis of classifier. For Logistic Regression, the model itself is difficult to fit the high complexity data and easy to under fit. For Random Forest and Extra Trees, the ensemble learning model shows its strong reliability and stability.

### Additional evaluation based on miRNA-disease association prediction

**Fig. 3:**
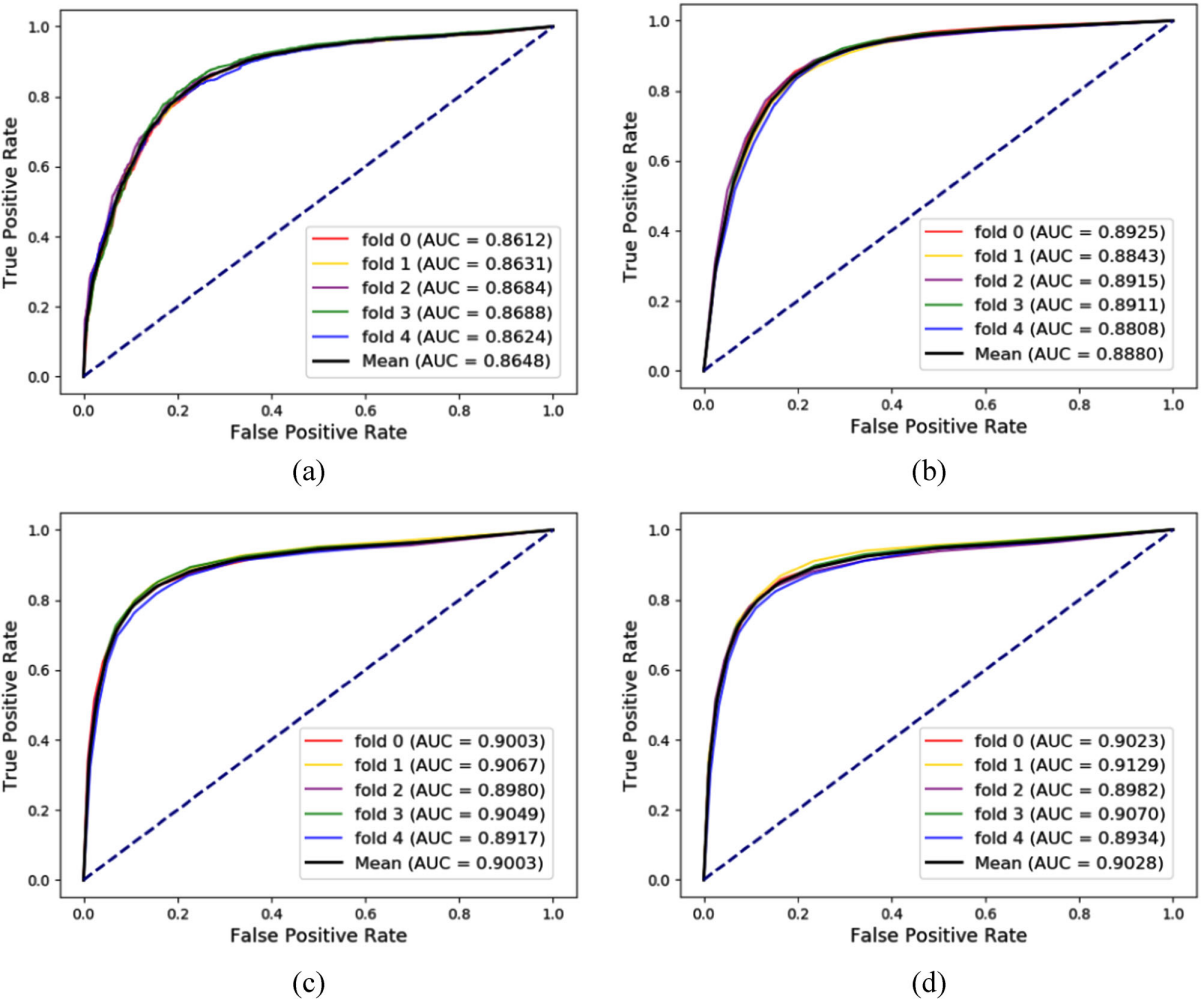
Comparison of the ROCs, AUCs, PRs, and AUPRs based on different methods. **a** All nodes are only represented by attribute information. **b** This is a traditional local method which measures behavior similarity by Gaussian kernel function. **c** This is a novel local method which measures behavior similarity by GF. **d** This is result of the global model presented in this paper which combines different kinds of relationships as many as possible.

Despite the emergence of many powerful prediction models as complements to manual experiments, the major limitation of the prediction capabilities of these methods is that the thoughts based on reductionism only considering the problem itself. In this section, we choose miRNA-disease association prediction as a more specific research object to compare the difference between the proposed global perspective method and the previous local point approach. After removing redundancy and uniform identifiers, 16427 miRNA-disease associations containing 901 different miRNAs and 877 different diseases were obtained from HMDD in April 26, 2019. The results of predicting miRNA-disease association in four different ways under 5-fold Cross-validation are shown in the Fig. 3.

In Fig. 3a, focusing on miRNA and disease, the nodes are only represented by attribute information, that is, miRNA sequence or disease semantics. This method is a baseline for comparison with other prediction models. In Fig. 3b, this is a traditional local method proposed by T. van Laarhoven et al.^22^, which is widely used in drug-target, miRNA-disease association prediction^23^. It is local method and measures the functional similarity of miRNA and disease by Gaussian kernel function. Briefly, 80% associations of the miRNA-disease network are processed by Gaussian kernel function in each fold and then each node can be represented as a 128-dimensional by combining node attributes and functional similarity vectors. In Fig. 3c, this is a local embedding method similar to the idea of Fig. 3b, 20% of miRNA-disease associations are used for test, and remaining 80% of the miRNA-disease associations are used for GF network embedding. Finally, each node is abstracted into a vector by concatenating node 2 kinds of feature. In Fig. 3d, this is result of the global model presented in this paper. In each fold of Cross-validation, 20% of miRNA-disease associations were segmented as the test sample, and the remaining miRNA-disease associations along with the rest eight kinds of associations were sent to GF for representation. Each node can be stated by combining node attributes and node behaviors. Competitive results relative to other methods demonstrate the superiority of the comprehensive and systemic perspectives to carry out this task.

In this section, by comparing proposed model with different previous methods, the results proved that additional relationships beyond direct research targets are valuable for predicting potential miRNA-disease associations. To our knowledge, the existence of information exchange between nodes in MAN provides a novel perspective to detect undiscovered associations in certain aspects and helps to recognize gene expression in a novel view.

### A case study based on miRNA-disease association

To evaluate the performance of our proposed model in a real environment, a case study of colon neoplasms was implemented on MAN-GF for yielding the most probable related miRNAs. First, pairs related to colon neoplasms were all removed from the dataset. Hence, the remaining 16282 (16427–145) miRNA-disease associations were used as training set to construct the model. Colon neoplasms connect all miRNAs to form association pairs respectively as the predictive sample. As a result, 18 of top-20 candidates were confirmed to have associations with colon neoplasms based on dbDEMC 2.0^24^ and HMDD^8^ as shown in the Table 5.

**Table 5:** MAN-GF was applied to Colon neoplasms to predict the potential disease-related miRNAs, and 18 of top-20 predicted miRNAs have been confirmed according to recent experimental literatures.

Num	miRNA	Disease	Evidence
1	hsa-mir-10a-5p	Colon neoplasms	dbDEMC 2.0
2	hsa-let-7b-5p	Colon neoplasms	HMDD/ dbDEMC 2.0
3	hsa-mir-183-5p	Colon neoplasms	dbDEMC 2.0
4	hsa-mir-431-5p	Colon neoplasms	dbDEMC 2.0
5	hsa-mir-136-5p	Colon neoplasms	dbDEMC 2.0
6	hsa-mir-155-5p	Colon neoplasms	HMDD/ dbDEMC 2.0
7	hsa-mir-324-5p	Colon neoplasms	dbDEMC 2.0
8	hsa-mir-454-5p	Colon neoplasms	dbDEMC 2.0
9	hsa-mir-29b-2-5p	Colon neoplasms	dbDEMC 2.0
10	hsa-mir-205-5p	Colon neoplasms	HMDD/ dbDEMC 2.0
11	hsa-mir-1-3p	Colon neoplasms	HMDD/ dbDEMC 2.0
12	hsa-mir-218-5p	Colon neoplasms	dbDEMC 2.0
13	hsa-mir-301a-5p	Colon neoplasms	dbDEMC 2.0
14	hsa-mir-494-5p	Colon neoplasms	dbDEMC 2.0
15	hsa-mir-376a-5p	Colon neoplasms	dbDEMC 2.0
16	hsa-mir-149-5p	Colon neoplasms	dbDEMC 2.0
17	hsa-mir-196a-1-3p	Colon neoplasms	unconfirmed
18	hsa-mir-4488	Colon neoplasms	unconfirmed
19	hsa-mir-199a-5p	Colon neoplasms	dbDEMC 2.0
20	hsa-mir-335-5p	Colon neoplasms	dbDEMC 2.0

## Conclusion

In this study, we constructed a large-scale Molecular Associations Network (MAN) including nine kinds of relationships among five types of biomolecules and a model called MAN-GF that can predict any links between arbitrary nodes in the framework. Specifically, MAN is a heterogeneous network with multiple biological elements consists various subnets including protein–protein interaction network, drug-target network, ncRNA-disease network and etc. Biomarker2vec focuses on learning the low-dimensional representation of nodes by attribute information and behavior information, which efficiently explains the intrinsic characteristic and topological properties of the complex network. MAN-GF made link prediction based on these projection vectors. Taking the mapped low-dimensional space vector as input, the Random Forest classifier is chosen to carry out the link prediction task. The proposed model achieved a competitive performance with AUC of 0.9647 and AUPR of 0.9521 under 5-fold Cross-validation, and additional experiments strongly support the existence of information transfer between biomolecules in MAN. In general, the proposed model can not only be treated as a supplement for wet experiments, but also stimulate researchers to step out in understanding the transmission of information between biomolecules. Although far from complete, the seamless integration of complex network technology with biological big data provides a new insight into the understanding of life activities and disease mechanisms at global view. We hope that this work can represent an important step towards a systematic and comprehensive perception in comprehension of all aspects in both computer and life sciences.

## Methods

### Materials

**Fig. 4:**
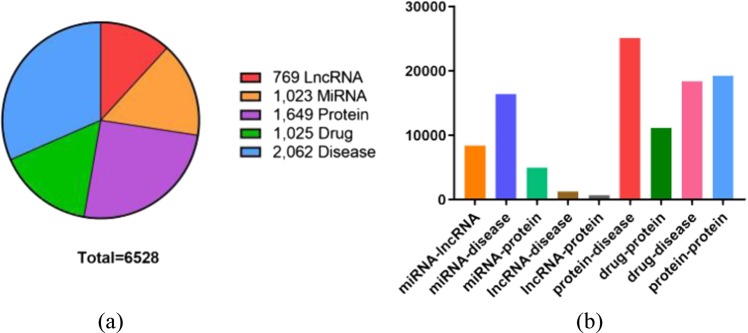
The statistics of biomolecules and relationships in the Molecular Associations Network (MAN). MAN is a heterogeneous attribute network which contains nine kinds of relationships among five types of biomolecules including miRNA, lncRNA, protein, drug, and disease. Figure 4a is the specific number of 5 biomolecules, and Figure 4b is the specific number of 9 relationships.

The known relationships and biomolecules are downloaded from various databases and carefully preprocessed^8–10,25–30^. The identifiers of miRNA, lncRNA, protein, and drug are unified by miRBase, NONCODE, STRING, and DrugBank, respectively. We directly applied the disease name of each original database. After the operations such as unified identifier, de-redundancy and removing nodes with low frequency just like described in the article of Zhang et al.^31^, a comparatively dense adjacency matrix with 6528 rows and 6528 columns was constructed by 105546 relationships to store the whole information of MAN. The details of the data can be seen in the Table 6 and Fig. 4. Besides, we uploaded all relationships on github pages: https://github.com/CocoGzh/MAN-1.0.

**Table 6:** The details of nine kinds of relationships.

Relationship type	Database	Number of pairs
miRNA-lncRNA	lncRNASNP2	8374
miRNA-disease	HMDD	16,427
miRNA-protein	miRTarBase	4944
lncRNA-disease	LncRNADisease lncRNASNP2	1264
lncRNA-protein	LncRNA2Target	690
protein-disease	DisGeNET	25,087
drug-protein	DrugBank	11,107
drug-disease	CTD	18,416
protein–protein	STRING	19,237
Total	MAN	105,546

In this paper, all experimentally validated biomolecule relationships (105,546 pairs) are treated as the golden standard positive dataset, and the same number unconfirmed relationships are randomly selected as the negative samples. Considering that there may exist potential true positive relationships in negative sample which are only a small part of the total number, the noises presented do not cause large deviations in the classifier. This is a typical technique for subsampling in unbalanced data and is widely used in bioinformatics and link prediction^32,33^.

### Node attribute representation: K-mer, semantic, and fingerprint

For miRNA, lncRNA and protein, sequences of them are downloaded from miRbase^34^, NONCODE^35^, and STRING^9^, respectively. The method proposed by Shen et al. is applied to analyze and normalized the components to characterize the sequence for both ncRNA and protein^36^. For ncRNA, the sequence consists of four types of nucleotides: A, C, G, and U. Each dimension of ncRNA feature vector represents the normalized frequency of the corresponding conjoint k, i.e., k-mer. In this paper, k is set to 3. Thus, each ncRNA sequence can be represented as a 64 (4 × 4 × 4) dimensional vector, where each element of the vector corresponds to the normalized frequency of the corresponding 3-mer in the sequence.

For protein, 20 kinds of amino acids are classified into four groups according to the polarity of the side chain including (Ala, Val, Leu, Ile, Met, Phe, Trp, Pro), (Gly, Ser, Thr, Cys, Asn, Gln, Tyr), (Arg, Lys, His), and (Asp, Glu). Then each protein sequence can be extracted by the above-mentioned ncRNA-like coding method through using the 4-letter reduced alphabet. Similarly, each protein sequence can be represented as a 64 (4 × 4 × 4) dimensional vector, where each dimension is a normalized component of each class of amino acids. Through the above sequence encoding operation, both ncRNA and protein can be represented as 64-dimensional vectors in preparation for subsequent node representation.

For disease, their Medical Subject Headings (MeSH) descriptors are downloaded from https://www.nlm.nih.gov/. MeSH is a standard vocabulary developed by the U.S. National Library of Medicine to index the magazines, journals, and terminology in the fields of biology and medicine. Previous literature points out that it is feasible to describe the similarities between diseases by MeSH Tree Structures and treat them as representation vectors^37^. A Directed Acyclic Graph (DAG) can be constructed by the descriptors of the disease, and the similarity between two diseases can be calculated based on the generalized Jaccard formula, i.e., the larger the intersection, the greater the similarity. The detailed description of the DAG is as follows: *DAG(D)* = *(D, N(D), E(D))*, *N(D)* is the point set that contains all the diseases in the *DAG(D)*. *E(D)* is the edge set that contains all relationships between diseases in the *DAG(D)*. The contribution of disease *t*, which is in the point set *N(D)* to the semantic value of disease *D* can be defined according to Eq. (1).

1 $$\left\{ {\begin{array}{*{20}{cc}} {D1_D\left( t \right) = 1}\hfill	 {\quad if\;t = D} \\ {D1_D\left( t \right) = \max \left\{ {\Delta \, {*} \, D1_D\left( {t^\prime } \right)|t^\prime \in {\mathrm{children}}\;{\mathrm{of}}\;t} \right\}}\hfill 	\quad{if\;t \, \ne \, D} \end{array}} \right.$$

where Δ denotes an attenuation factor. In the DAG generated by disease *D*, *D*’s contribution to itself can be regarded as the maximum and equals to 1, and the remaining diseases will contribute less and less to disease *D* as the distance increases. Therefore, the sum of the contributions of diseases, which are in the set *N(D)* to *D* can be calculated according to Eq. (2).

2 $$DV1\left( D \right) = \mathop {\sum}\limits _{t \in N\left( D \right)}D1_D\left( t \right)$$

Then the similarity between diseases *i* and *j* can be calculated according to Eq. (3).

3 $${\mathrm{Similarity}}\left( {i,j} \right) = \frac{{\mathop {\sum}\nolimits_{t \in N\left( i \right) \cap N\left( j \right)} {\left( {D1_i\left( t \right) + D1_j\left( t \right)} \right)} }}{{DV1\left( i \right) + DV1\left( j \right)}}$$

Where *DV*1(*i*) and *DV*1(*j*) are the sum of the contributions of disease in *N*(*i*) and *N*(*j*) to *i* and *j*. *N*(*i*) ∩ *N*(*j*) is the intersection of *N*(*i*) and *N*(*j*)*. D*1_*i*_(t) and *D*1_*j*_(*t*) is the disease value of *t* to *i* and *j* in *N*(*i*) and *N*(*j*), respectively.

The attribute information of disease can be represented by disease semantic similarity, which is converted into a 64-dimensional vector after feature extraction and transformation by the sparse autoencoder.

For drug, we download drug SMILES from DrugBank and then transform them into Morgan Fingerprint by python package. In order to reduce noise and improve feature quality, sparse autoencoder is used to obtain the appropriate feature space from the original space.

### Node behavior representation: Graph Factorization

Obviously, the adjacency matrix contains all the content of the network, where the *i*-th row can be considered as the one-hot representation vector of the *i*-th node. Although this kind of sparse representation can include all the node behavior information and is beneficial to the design of the discrete algorithm, it is not friendly to the storage and the construction for downstream classifier. We hope to abstract the nodes into vectors through associations in a simple and efficient way. In this paper, an algorithm called Graph Factorization (GF), which first obtains a graph embedding in $O\left( {\left| E \right|} \right)$ time^38^ is applied to carry out this task. To achieve this goal, GF factorizes the adjacency matrix of the graph, minimizing the loss function according to Eq. (4).

4 $$f\left( {Y,Z,\lambda } \right) = \frac{1}{2}\mathop {\sum}\limits_{\left( {i,j} \right)\upepsilon E} {( {Y_{ij}\, -\, < Z_i,Z_j > } )^2} + \frac{\lambda }{2}\mathop {\sum}\limits_i {\left\| {Z_i} \right\|^2}$$

Where *Y* is the weight adjacency matrix and *Z* is the factor matrix. *λ* is regularization parameter. *E* is the edge set, and *i* and *j* are edges in *E*.

The gradient of *f* with respect to the row *i* of *Z* can be given according to Eq. (5).

5 $$\frac{{\partial f}}{{\partial Z_i}} = - \mathop {\sum}\limits_{j \in N\left( i \right)} {( {Y_{ij}\, -\, < Z_i,Z_j > } )Z_j + \lambda Z_i}$$

Where *N*(*i*) is the set of neighbors of node *i*.

For a pair $\left( {i,j} \right) \in E$ this amounts to Eq. (6).

6 $$( {Y_{ij}\, -\, < Z_i,Z_j > } )Z_j + \lambda Z_i$$

Stochastic gradient descent is a common way of solving this nonconvex problem and algorithm is as follows:

**Table:** 

Algorithm: Sequential stochastic gradient descent
**Require:** Matrix $Y \in R^{n \times n}$, rank *r*, accuracy ${\it{\epsilon }}$
**Ensure:** Find a local minimum of equal (4)
(1) Initialize $Z^\prime \in R^{n \times r}$ $\in$ at random
(2) $$t \, {\leftarrow} \, 1$$
(3) **repeat**
(4) $$Z^\prime \, {\leftarrow} \, Z$$
(5) **for all** edges $\left( {i,j} \right) \in E$ **do**
(6) $${\boldsymbol{\eta }} \leftarrow \frac{1}{{\sqrt {\it{t}} }}$$
(7) $$t \, {\leftarrow} \, t +1$$
(8) $$Z_i \leftarrow Z_i + {\boldsymbol{\eta }}[(Y_{ij}\, -\, < Z_i,Z_j > )Z_j + \lambda Z_i]$$
(9) **end for**
(10) **until** $$\left\| {Z - Z^\prime } \right\|_{Frob}^2 \le {{\upepsilon }}$$
(11) **return Z**

Note whenever the representation of the node is embedding via GF, the tested links are stripped to ensure that the label information is not leaked into the test set. Given the actual situation such as the new sample problem, etc., the degree of each node is not guaranteed to be greater than 0 when segmenting the dataset.

### Sparse autoencoder

In view of the large quantity and multi-dimension of positive and negative samples produced, it is not conducive to the model construction. Sparse Autoencoder (SAE) is mainly utilized for vector reconstruction to unify dimension. SAE is an unsupervised feature learning algorithm which aims to learn a high-level structured representation from original feature space. SAE can be divided into two parts: the encoder that encodes the input data into corresponding representation *h* and the decoder that reconstructs an approximation *x̂* from the hidden representation *h*. In general, the function of SAE is to extract and transform features by minimizing the error between input and output with backpropagation algorithm. The cost function of Autoencoder can be defined according to Eq. (7).

7 $$J\left( {W,b} \right) = \frac{1}{m}\mathop {\sum}\limits_{i = 1}^m {\frac{1}{2}\left\| {x - \widehat x} \right\|^2}$$

where *m* is the number of training data, which can be defined according to Eq. (8).

8 $${\widehat {x}} = o^{\left( {n_l} \right)}$$

where *n*_*l*_ denotes the number of layers of the network, *o*^(*nl*)^ is the output of the *n*_*l*_-layer, which can be defined according to Eq. (9).

9 $$o^{\left( {n_l} \right)} = f\left( {o^{\left( {n_l - 1} \right)} + b} \right)$$

where *b* is the threshold of neurons and *f* is the activation. Relu activation function is chosen to perform this operation, which can be defined according to Eq. (10).

10 $$f\left( x \right) = \max \left( {0,x} \right)$$

The cost function of SAE comprises three terms can be defined according to Eq. (11).

11 $$J\left( {W,b} \right) = \frac{1}{m}\mathop {\sum}\limits_{i = 1}^m {\frac{1}{2}\left\| {x - \widehat {x}} \right\|^2} + \alpha \mathop {\sum}\limits_{j = 1}^n {KL\left( {\rho \parallel \widehat \rho _j} \right) + \beta \left\| w \right\|_2^2}$$

The first part is to describe the error between input *x* and output $\widehat x$, In the second part, *n* indicates the number of hidden layer units. The average activity of hidden neurons can be described by KL divergence and limits the loss function. The third part is the weight decay term, the purpose of which is to reduce the magnitude of the weight and prevent over-fitting.

### Statistics and reproducibility

The collection details of the biomolecule relationships are described in the material section. After uniform identifiers and de-redundancy, the whole network is constructed. Data used for analysis are available on GitHub page: https://github.com/CocoGzh/MAN-1.0. All experiments are implemented under Python 3.7 and all paraments are set to default value. In addition, thanks for the python package Numpy 1.16.4, Scikit-learn 0.21.2, Tensorflow 1.14.0, Keras 2.2.5, and Open-NE.

### Reporting summary

Further information on research design is available in the Nature Research Reporting Summary linked to this article.

## Supplementary information

Reporting Summary

## Data Availability

The data that support the findings of this study are available from the corresponding author on request, or the data for analysis can be accessed in GitHub page: https://github.com/CocoGzh/MAN-1.0

## Code availability

The code developed can be accessed in GitHub page: https://github.com/CocoGzh/MAN-1.0
